# One day you'll wake up and won't have to go to work: The impact of changes in time use on mental health following retirement

**DOI:** 10.1371/journal.pone.0199605

**Published:** 2018-06-28

**Authors:** Tim Olds, Nicola W. Burton, Judy Sprod, Carol Maher, Katia Ferrar, Wendy J. Brown, Jannique van Uffelen, Dorothea Dumuid

**Affiliations:** 1 Alliance for Research in Exercise, Nutrition and Activity (ARENA), University of South Australia, Adelaide, Australia; 2 Murdoch Children’s Research Institute, Melbourne, Australia; 3 School of Human Movement and Nutrition Sciences, University of Queensland, Brisbane, Australia; 4 School of Applied Psychology, Griffith University, Brisbane, Australia; 5 University of Leuven, Department of Movement Sciences, Leuven, Belgium; Charite Medical University Berlin, GERMANY

## Abstract

**Background:**

Retirement is a life transition involving an obligatory change in how people use their time. Because there are strong associations between use of time and health, different changes in time use following retirement may have different impacts on mental health.

**Methods:**

105 participants were followed from 6 months before retirement to 12 months after retirement. At each time-point, use of time was quantified using a validated computerised 24-hour recall. Depression, anxiety and stress were assessed using the Depression, Anxiety and Stress Scales (DASS21), well-being with the Short Warwick-Edinburgh Mental Well-being Scale (SWEMWBS), life satisfaction with the Australian Unity Personal Well-being Index (AUPWI), and self-esteem with the Rosenberg Self-Esteem Scale. Time-use data were analysed using compositional data analysis, which treats the 24-h day as a holistic “activity composition” rather than as individual activity domains. Time flow analytics were used to map patterns of change in time use from pre-retirement to post-retirement. Regression analysis was used to determine whether changes in the activity composition were significantly associated with changes in mental health. Compositional isotemporal substitution models were used to illustrate dose-response relationships between changes in time use and conditional changes in mental health for individual activity domains, such as sleep, screen time and physical activity.

**Results:**

Following retirement, time no longer spent in work flowed mainly to household chores, sleep, screen time and quiet time (e.g. reading). Mental health improved overall. Changes in the activity composition were significantly related to conditional changes in DASS21 total score, depression, stress, and self-esteem, but not to anxiety, well-being or life satisfaction. Replacing work time with physical activity or sleep was associated with positive changes in mental health. Effect sizes for 60-minute substitutions ranged from –0.15 to +0.31.

**Conclusion:**

Following retirement, replacing work with physical activity, and to a lesser extent sleep, is associated with better mental health.

## Background

Life transitions such as moving from education to work, or having children, involve an obligatory re-organisation of the way we use our time. Retirement is one such life transition. People fill in the hours previously devoted to work in different ways, partly through choice and partly through obligation. Because the way we use our time profoundly affects our health, different changes in time use upon retirement may affect both mental and physical health in different ways.

Mental health may be particularly sensitive to change at retirement, and research in this area provides mixed evidence. Some studies have shown statistically significant associations between retirement and decreased life satisfaction and greater psychological distress, while others have found either no deleterious, or positive effects [1]. In some cases retirees may feel more energetic and satisfied as they explore new activities and roles. Reduced job stresses and time pressure may be beneficial, as indicated by higher morale during the retirement transition in one US study, perhaps moderated by factors such as sex and socio-economic status [1]. Retirement is associated with fewer symptoms of depression among people who have high levels of work stress interfering with family activities before retirement [2]. Retirement could also precipitate positive or negative changes in identity, social interaction and intellectual activities, as well as in financial security and family relationships, all of which are known to impact on mental health [3]. Research from the USA and Germany has suggested that the large majority of retirees experience minimum changes in mental health, some experience negative changes during the initial transition but then improvements, and only a small proportion experience positive changes [4].

Specific health-related behaviours may change after retirement and mediate changes in mental health. There is strong evidence that being physically active is associated with mental health benefits in otherwise healthy adults. The evidence is strong for ameliorative and protective effects against depression [5] and for enhancement of quality of life (e.g., vitality) [6], and positive but weaker effects for management of and protection against anxiety and stress [7]. There is evidence to suggest that duration, quality and timing of sleep are also associated with mental health [8]. It is therefore reasonable to assume that, if people were to substitute time at work for more time in physical activity and sleep after retirement, there may be positive changes in indicators of mental health.

Capturing and accurately representing time use across the range of daily activities presents substantial methodological challenges. While objective measurement using accelerometry will capture time spent in various activity intensity bands, it cannot as yet capture the type of activity people are doing. Questionnaires and surveys about individual activity domains such as physical activity have poor validity [9]. Many questionnaires tend to focus only on one type of behaviour, such as sleep, physical activity, or screen time, which constrains assessment of potential substitution effects. Time-use recalls offer a promising alternative, providing both good validity and high resolution [10], as well as information about a range of types and contexts of daily activities across different life domains.

An important statistical and conceptual challenge is the compositional nature of time use. Our days are made up of various activities which can be categorised into domains, such as work or household chores, which are mutually exclusive and exhaustive. A corollary is that they are co-dependent: because there are only 24 hours in a day, any change in time spent in one domain must be compensated by equal and opposite net changes in other domains. As a result, we cannot use standard statistical models which incorporate all time-use domains, because they are perfectly multi-collinear [11]. Even when a “leave-some-out” approach is used, spurious associations can arise due to co-dependency [12]. It is also theoretically impossible to know whether associations between a given time-use domain, such as screen time, and a given outcome, such as adiposity, are due to more screen time or less of whatever screen time displaces, for example sleep or physical activity. A conceptual solution for this dilemma is the application of compositional data analysis (CoDA) to time-use data [13]. CoDA analyses use of time data in terms of ratios of time spent in combinations of domains, and requires a non-Euclidean native geometry called the Simplex [14]. Outputs can be mapped onto Euclidean space, allowing standard statistical techniques such as regression to be used. A compositional variant of isotemporal substitution [15] can then be used to model the effects of displacing time spent in a given activity domain with time spent in another domain, or combination of domains.

The aims of this study were to describe changes in time use across the retirement transition; to determine whether these changes were associated with changes in mental health; and to describe the relative effects of replacing work time with time from other activity domains, such as physical activity, sleep and screen time. Time use was captured using a valid, reliable, high-resolution use-of-time recall, and changes in time use were modeled using time flow analytics and CoDA.

## Methods

### Participants

The participants in this study were from a cohort study of Australian retirees, the Life After Work (LAW) study [16], that recruited via advertisements, word of mouth, presentations at a retirement planning seminars, and a recruitment company. Ethics approval for this study was provided by The University of South Australia HREC (Protocol no. 0000023494) and The University of Queensland Behavioural and Social Sciences ERC (Protocol no. 2011001152). Written informed consent was obtained from all participants. Data on use of time, physical and mental health, and socio-demographic characteristics were gathered 6 months prior to, and at 3, 6 and 12 months post-retirement. This study compares data from the pre-retirement and 12 months post-retirement time points.

### Measures of mental health and well-being, and covariates

At both time points, participants completed a battery of questionnaires assessing mental health and well-being. **Depression**, **anxiety** and **stress** were quantified using the Depression, Anxiety and Stress Scales (DASS21), a 21-item self report instrument with subscales to measure the three emotional state over the past week. The scales have established validity [17]. Subscales scores range from 0 to 42, with higher scores indicating poorer mental health. Although not used in this study, data can be categorized to indicate severity, with “mild” severity indicated by scores of 10–13 for depression, 8–9 for anxiety, and 15–18 for stress; and “moderate” severity indicated by scores of 14–20 for depression, 10–14 for anxiety, and 19–25 for stress [18]. This study also used the overall score of general distress, which ranges from 0–126. **Well-being** was assessed using the Short Warwick-Edinburgh Mental Well-being Scale (SWEMWBS), a 7-item questionnaire assessing positive mental health over the last week. The scale shows adequate internal consistency and reliability and acceptable construct, criterion-related, and discriminant validity [19]. **Life satisfaction** was evaluated using the Australian Unity Personal Well-being Index (AUPWI), an 8-item questionnaire measuring satisfaction with life as a whole, as well as standard of living, health, achievement in life, personal relationships, safety, feeling part of the community, security and spirituality/religion. The AUPWI has good test-retest reliability and convergent validity [20]. **Self-esteem** was quantified using the Rosenberg Self-Esteem Scale, a 10-item questionnaire with strong test-retest reliability and established concurrent, predictive and construct validity [21].

### Covariates

In addition, participants were asked about their marital status, educational qualifications, job descriptions, reasons for retirement, and gross **household income** using items from the Australian Bureau of Statistics census, and their health was assessed using the Short-Form Health Survey (SF36) [22]. This 36-item survey assesses several domains of health, but only the **Role limitations** (**physical)** subscale was used in this study as a covariate to adjust for changes in activities due to physical health. The Role limitations (physical) subscale of the SF-36 has good test-retest reliability (r = 0.69) and high internal consistency (alpha = 0.96) [23].

### Time-use-recalls

Participants recalled time use with the Multimedia Activity Recall for Children and Adults (MARCA) [24]. The MARCA uses the day reconstruction technique, in which participants were “walked through” the previous two days from midnight to midnight, recalling each activity chronologically. They could choose from over 500 different activities in time slices as fine as 5 minutes. Each MARCA activity is linked to an energy expenditure compendium [25] so that time spent in various energy expenditure bands [sleep (<1 MET); sedentary behaviour (1–1.5 METs, standing or reclining; light physical activity (1.6–2.9 METs); moderate-to-vigorous physical activity (≥3 METs)] can be calculated. The adult version of the MARCA has very high same-day test-retest reliability (ICC = 0.990–0.997) and good validity compared to accelerometry (rho = 0.72; [26]) and doubly labelled water (rho = 0.70; [27]). The MARCA was administered at each time point on two occasions, each time recalling the two previous days. The four-day sample in all but four cases included at least one weekday and one weekend day at both baseline and follow-up. MARCA activities are hierarchically aggregated into 9 “superdomains” (Table 1). Time spent in each superdomain was averaged across the four days, using a 5:2 weighting for weekdays:weekend days.

**Table 1:** The 9 MARCA superdomains.

Superdomain	Description	Examples
Chores	Indoor and outdoor household chores	Washing dishes Gardening
Physical Activity	Exercise and sport	Gym work Playing tennis
Quiet Time	Reading and other quiet pursuits	Reading a book Listening to music
Screen Time	Using small and large screens (except for work)	Watching TV Playing computer games
Self-care	Eating and grooming	Eating dinner Showering
Sleep	Sleeping and napping	
Social	Socialising and cultural activities	Family get-togethers Arts and crafts
Transport	Active and passive transport	Walking to the shops Driving a car
Work	Paid and unpaid employment and study	Office work Typing on a computer

### Time flow analytics

Time flows among superdomains were calculated by comparing each 5-minute time slice at pre-retirement with the corresponding time-slice on a randomly chosen day for the same participant matched for day type (i.e. week day or weekend day) at 12 months post-retirement. Any change in superdomain was added to the time flow. For example, if at 1 pm on a weekday at baseline the participant was engaged in filing (Work), and at 1 pm on a matched weekday at follow-up she was engaged in reading (Quiet Time), 5 min was added to the time-flow from Work to Quiet Time. Time flows were calculated for all participants. Time flows were then weighted 5:2 for weekdays: weekend days, and averaged across all participants. Net time flows were calculated by subtracting the time flows out of a superdomain to another given superdomain from the corresponding time flows in. Chord diagrams drawn using the R *circlize* package [28] were used to graphically represent time flows.

### Compositional data analysis

Compositional data analyses were performed in R [29] using the *Compositions* [30] and *zCompositions* [31] packages. Each participant’s daily time use was conceptualized as a composition with the average daily duration of all time-use domains summing to 1440 minutes (24 hours). Following the principles of CoDA, compositions were expressed as isometric log ratio *(ilr)* coordinates systems [32]. The *ilr* coordinates isometrically map the compositions in real space; all relative information about the compositional parts is preserved in the set of *ilr* coordinates. To enable the application of *ilr* coordinates, zero values in any time-use domain were replaced by very small values: 65% of the 5-minute sampling frame [33]. Once the compositions were expressed in real space as *ilr* coordinates, standard statistical methods were applied as described below.

Change in time-use composition was calculated for each participant by finding the difference between the *ilr* coordinates before retirement and 12 months after retirement. This was used as the explanatory variable in a multiple linear regression model. Changes in each of the outcome variables (DASS21 total score, depression, anxiety, stress, well-being, satisfaction and self-esteem) were considered as dependent variables, adjusted for pre-retirement values. Changes in physical role limitations and household income were included as covariates. The relationship between change in time-use composition (expressed as a set of *ilr* coordinates) and change in outcome was assessed by MANOVA contrast with type II test, i.e., testing the influence of each variable adjusted for all other variables.

### Compositional isotemporal substitution

The multiple linear regression models for conditional change in each outcome variable were used to predict mental health for a series of systematically altered compositions. Specifically, predictions were made for change-in-time-use compositions (as *ilr* coordinates) where fixed durations of work had been replaced by other time-use domains. The predictive change-in-time-use compositions were systematically constructed by finding the difference between the mean time-use composition before retirement and the post-retirement composition, where a set duration of time had been reallocated from work to one other time-use domain, keeping the remaining time-use domains constant at their mean values. The procedure was repeated to create predictive change compositions representing the reallocation of time from work to each of the other time-use domains. Subsequently, the predictive change compositions, expressed as *ilr* coordinates, were used to calculate the change in outcome for all possible reallocations of fixed durations of time from work, in 10-minute increments from 10 to 60 minutes. Finally, to isolate the predicted change in outcome associated with the change-in-time-use composition, predicted change in outcome for no change in time-use composition (as *ilr* coordinates) was subtracted from the original change predictions. The predicted change in outcomes for isotemporal substitution of 0 to 60 minutes of work for other time-use domains were plotted as effect sizes relative to the pooled pre- and post-retirement values to aid visualisation of the relationships.

### Power analysis

Alpha was set at 0.05, but sequential Bonferroni adjustment was used to allow for multiple comparisons across the seven mental health outcomes. With a sample size of 101, 3 predictor variables, and a power of 0.8, this design is capable of detecting effect sizes of r≥0.35.

## Results

### Socio-demographic characteristics of the sample

A total of 139 participants recalled their use of time at baseline, and 116 at 12 months post-retirement. Of these, 11 did not provide income data at either baseline or follow-up, and were excluded from analysis, leaving a final sample of n = 105. The excluded participants did not differ from the retained sample in age, sex, baseline income or any baseline mental health score; however they had a higher mean body mass index (p = 0.02).

The pre- and post-retirement characteristics of the final sample are shown in Table 2. Two-thirds of the final sample (n = 68) were married or in a de facto relationships; the remainder were single, widowed or divorced/separated. Of those with partners, 55% had partners who had already retired. Over half (n = 54) had university qualifications, 28 had some post-secondary education, and 19 had only completed high school. Over half (n = 55) were professionals, such as teachers, or associate professionals, such as technicians, 28 had clerical positions, and 16 were managers. The average self reported time spent working before retirement was 34.3 (SD 8.6) hours/week. Following retirement, half (n = 50) continued to do some part-time work, averaging 4 (range 1–10.5) hours/week. the most common reasons for retirement were desire for a different life (n = 94), financial advantages (n = 62), having reached the age of retirement (n = 43), job stress (28), partner retirement (n = 19), carer duties (n = 18), and being retrenched (n = 12).

**Table 2:** Socio-demographic, health and time-use characteristics of the final sample (n = 105) before and after retirement. Values shown are means (SDs). Pre- and post-retirement values were compared using dependent t-tests. Significant differences are highlighted in **bold**.

		Pre-retirement	Post-retirement	P
Socio-demographic	Age	62.3 (4.3)	63.4 (4.3)	
	Sex (n, % female)	54 (51.4%)		
	BMI (kg.m^–2^)	27.1 (4.1)	27.1 (4.2)	0.34
	Income (AUD k)	93.9 (44.3)	60.1 (39.1)	**<0.0001**
	SF-36 Role limitation (Physical)	83.1 (31.8)	84.3 (31.4)	**0.0001**
Mental health	DASS21 total score	7.1 (6.1)	4.9 (6.9)	**<0.0001**
	DASS21 depression	2.0 (2.6)	1.3 (2.4)	**0.005**
	DASS21 anxiety	1.2 (1.6)	0.9 (1.9)	0.06
	DASS21 stress	3.9 (3.2)	2.6 (3.3)	**0.0001**
	SWEMWBS well-being	24.7 (3.8)	26.6 (4.7)	**<0.0001**
	AUPWI life satisfaction	7.8 (1.3)	8.2 (1.6)	0.10
	Rosenberg self-esteem	24.1 (4.7)	25.1 (4.6)	**0.01**
Time use	Chores (min/day)	162 (72)	216 (78)	**<0.0001**
	PA (min/day)	14 (26)	22 (41)	**0.03**
	Quiet Time (min/day)	74 (49)	90 (62)	**0.007**
	Screen Time (min/day)	117 (72)	149 (100)	**<0.0001**
	Self-care (min/day)	118 (31)	128 (34)	**0.002**
	Sleep (min/day)	462 (51)	493 (63)	**<0.0001**
	Social (min/day)	133 (71)	128 (69)	0.53
	Transport (min/day)	135 (52)	110 (50)	**0.0002**
	Work (min/day)	224 (93)	102 (81)	**<0.0001**

AUD = Australian dollars; AUPWI = Australian Unity Personal Well-being Index; BMI = body mass index; DASS21 = Depression, Anxiety and Stress Scales-21; PA = physical activity; SF-36 = Short Form Health Survey; SWEMWBS = Short Warwick-Edinburgh Mental Well-being Scale

### Changes in mental health

Mental health generally improved across the retirement threshold, with significant reductions in DASS21 total score, depression, and stress (all p≤0.0001), and improvements in well-being (p<0.0001) and self-esteem (p = 0.01). Life satisfaction and anxiety did not change significantly. There were no significant differences in changes in any of the mental health variables (p = 0.46–0.98) between men and women, although the study was underpowered to detect sex differences.

### Changes in activity composition across the retirement threshold

Participants reduced their work time by an average of 122 min/day. While a reduction of 122 min/day in work may seem modest after retirement, it should be remembered that this change is averaged over all days of the week, that not all participants were working full-time at pre-retirement, that some continued part-time work after retirement, and that some work-related activities are captured in other superdomains, such as Social (for example, lunch meetings). There was also a significant reduction of time spent in Transport (–25 min/day, p = 0.0002). The reductions in Work and Transport time were made up by significant increases in Chores (+54 min/day, p<0.0001), Screen Time (+32 min/day, p<0.0001), Sleep (+31 min/day, p<0.0001), Quiet Time (+16 min/day, p = 0.007), Self-Care (+10 min/day, p = 0.002), and Physical Activity (+8 min/day, p = 0.03). In terms of energy expenditure bands, participants decreased time spent in sedentary behaviours (–51 min/day), which offset increases in time spent asleep (+33 min/day), in light PA (+14 min/day), and in moderate-to-vigorous PA (+4 min/day). There were no significant differences in changes in any of the time-use variables (p = 0.58–0.96) between men and women.

### Time flows

**Fig 1:**
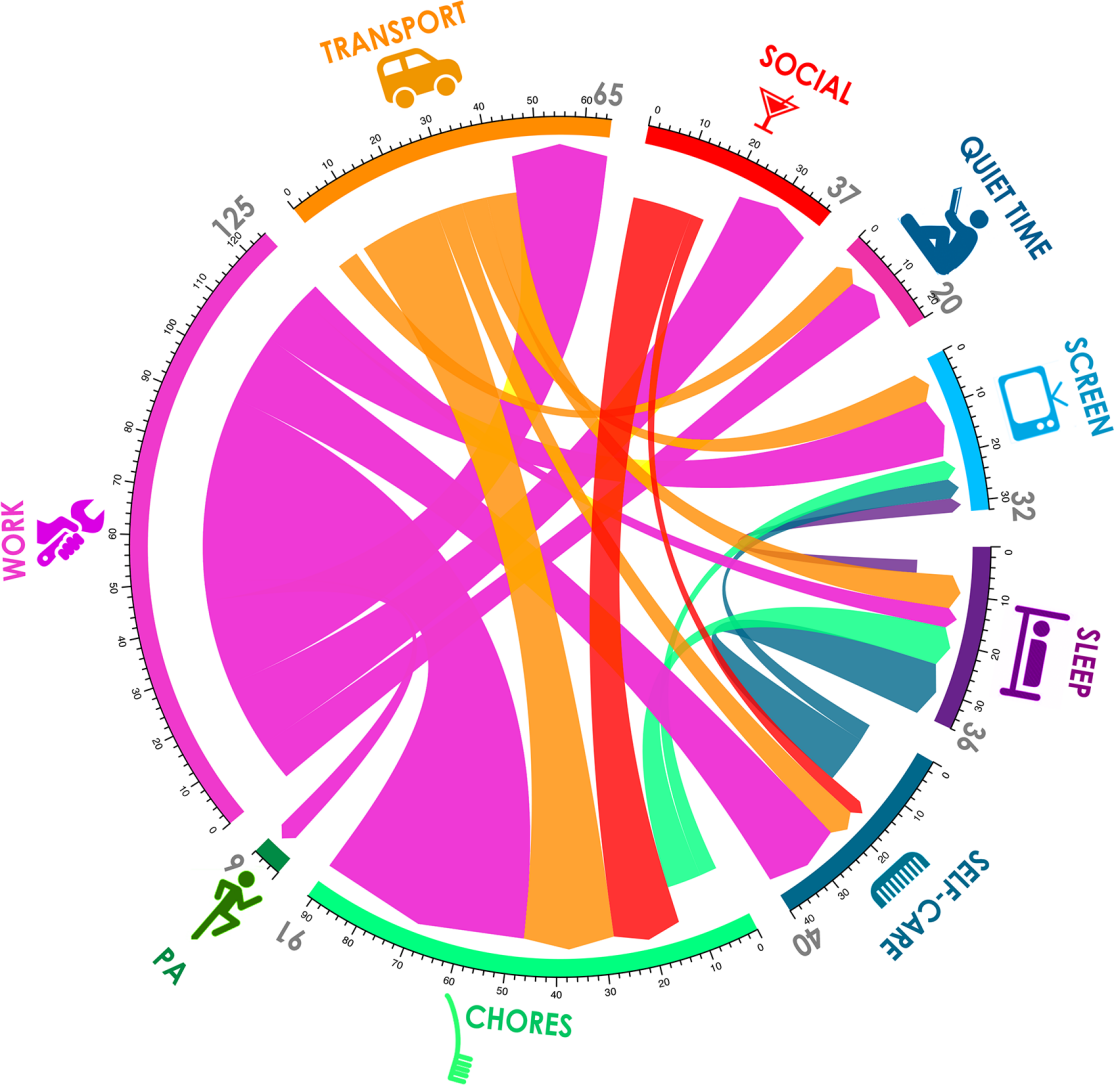
Chord diagram of the net superdomain time flows. The time around the circumference of the circle represents the total time flux (in and out) for each superdomain. The arrowheads represent the direction of the flow. For example, a total of about 90 min/day flowed in and out of the Chores superdomain. Most flowed in from Work, Transport and Social, but there was a small outflow to Screen Time and Sleep.

Time flow analysis showed a similar pattern. Fig 1 shows a chord diagram of the net superdomain time flows. The broad picture is of a time flow out of Work into a variety of superdomains such as Chores and Transport. An average of 125 min/day flowed from Work, mainly to Chores (+44 min/day), Transport (+20 min/day), Self-care and Social (+16 min/day each), and Screen Time (+12 min/day). Time devoted to Chores increased by 63 min/day, mainly due to net flows from Work (–44 min/day), Transport (–19 min/day) and Social (–14 min/day). Screen time increased by an average of 32 min/day, with Work (–12 min/day) being the biggest contributor. Transport decreased by 25 min/day, with flows going mainly to Chores (+19 min/day), but with an inflow of 20 min/day from Work, presumably reflecting the elimination of the journey to and from work. Sleep increased by 24 min/day, with the main contributor being Self-care (–11 min/day). Net time flows for Social, Quiet Time, Physical Activity and Self-care were <15 min/day.

### The relationship between changes in the activity composition and conditional changes in mental health

The change in overall activity composition was significantly related to changes in DASS21 total score (p = 0.006), depression (p = 0.03), anxiety (p = 0.26), stress (p = 0.02), and self-esteem (p = 0.02), but not to changes in well-being (p = 0.25) or life satisfaction (p = 0.91). Of these associations, however, only DASS21 total score remained significant after sequential Bonferroni adjustment.

**Fig 2:**
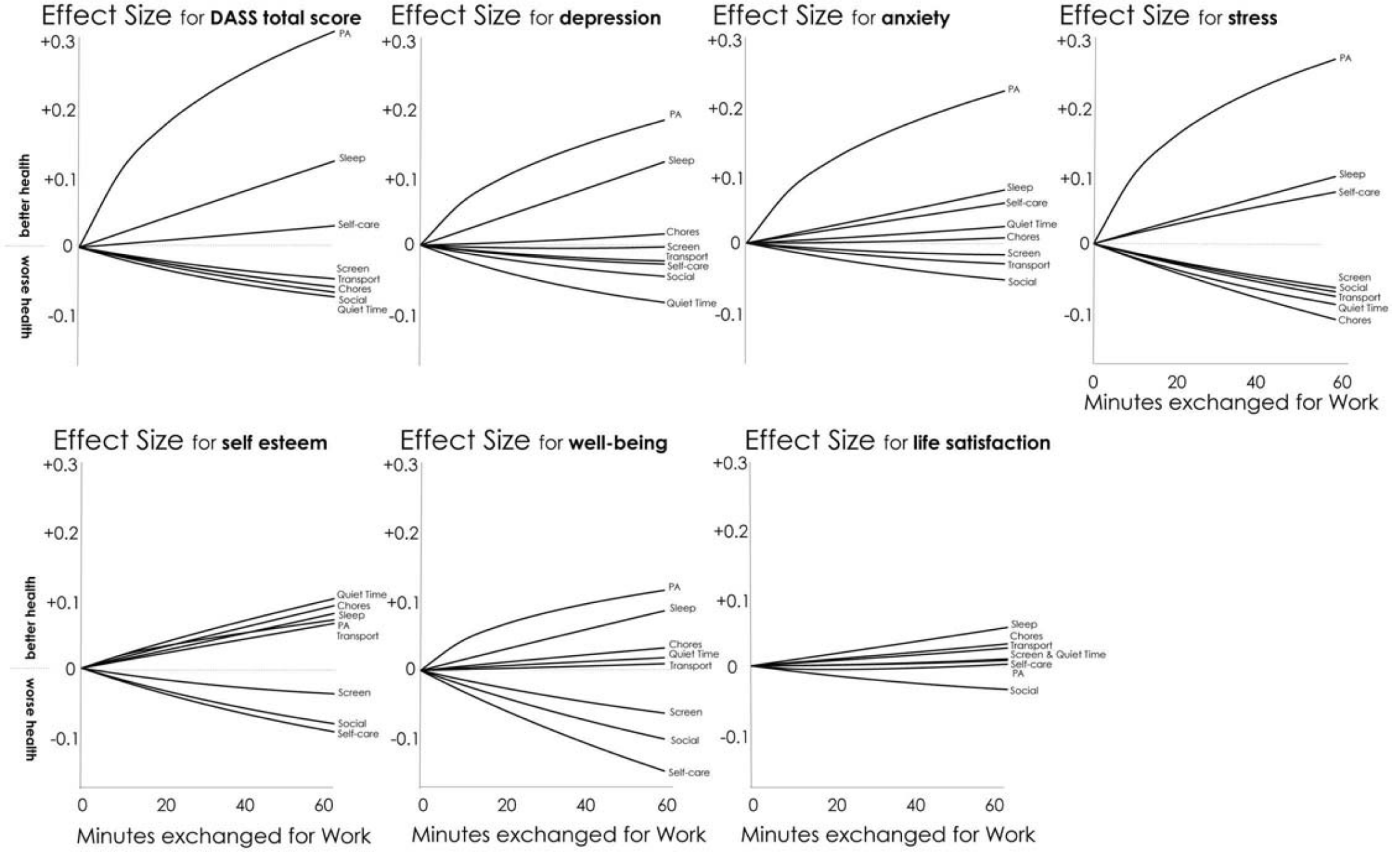
Modeled changes in mental health associated with substitution of 0–60 min/day from work to other superdomains. Changes are expressed as effect sizes relative to the pooled pre- and post-retirement values. PA = physical activity.

Compositional isotemporal substitution showed generally nonlinear changes in mental health as time spent in Work was replaced by time in other superdomains, with effect sizes (change scores relative to pooled baseline and post-retirement values) ranging from –0.15 to +0.31 for 60-min substitutions (Fig 2). The most favourable substitutions were replacing Work with Physical Activity or Sleep. Unfavourable substitutions showed lower effect sizes, and usually involved replacing Work with Screen Time or Social (Fig 2).

## Discussion

### Main findings

Following retirement, mental health significantly improved. About 40% of the time previously spent in work was replaced by household chores, and 20% each by sleep, screen time, and quiet time (e.g. reading). Importantly, changes in time use were significantly associated with changes in depression, stress and self-esteem, with small-to-moderate effect sizes. In particular, replacing work time with physical activity and sleep was always associated with improvements on all measures of mental health, while replacing work with screen time and social activities was always associated with decrements, albeit more modest.

The changes in use of time across retirement identified in this study are, by and large, consistent with those identified in previous studies. For example, both cross-sectional [34–36] and longitudinal [37] studies have shown increases in time spent in household chores post-retirement, particularly among men. In addition, Sprod et al’s [38] systematic review of changes in sedentary behaviour across retirement identified increased time spent in sleep and screen time.

The finding that mental health improved at retirement is consistent with the conclusions of van der Heide et al. (2013) [39], in their systematic review of 22 longitudinal studies. This does, however, contrast with some previous research. For example, Nordenmark and Stattin’s [40] analysis of the Swedish Panel Survey of Ageing and the Elderly found that retired people had higher levels of anxiety, distress and unhappiness compared with working people of a similar age. Dulin et al’s [41] study of over 6,000 New Zealand older adults found that retired participants had significantly poorer mental health than employed participants. The divergence in findings between these studies and the current study may be due to the cross-sectional nature of the Nordenmark and Stattin [40] and Dulin et al [41] studies. Of note, Nordenmark and Stattin [40] reported that 60% of their retired participants had done so due to ill health, thus it is possible that their poorer mental health and wellbeing preceded and contributed to their retirement, rather than being caused by it. Dingemans and Henkens (2015) [42] found involuntary retirement to be associated with decreases in life satisfaction, particularly if there was a lack of bridging work. Wang and Shi (2014) [4] concluded that retirees’ mental health did not follow a uniform pattern of transition and adjustment and is moderated by factors such as financial and physical wellbeing, as well as work and retirement characteristics.

The way in which use of time was reassigned across retirement was differentially associated with changes in mental health. In particular, increased time in physical activity was associated with substantial improvements in depression, anxiety and stress, and small improvements in self-esteem and wellbeing. This is consistent with experimental (e.g. [43]) and longitudinal evidence [44, 45] examining the relationship between physical activity and mental health outcomes in a range of adult populations. In our study, increased time sleeping was associated with small improvements in all mental health outcomes. This is consistent with findings from the US Behavioral Risk Factor Surveillance System suggesting that poor sleep is commonly experienced in the general population, and is associated with mental distress [46] and depression [47]. Other studies have highlighted a U-shaped relationship between sleep duration and mental health, with poorer outcomes associated with both short and long sleep duration [48, 49]. Finally, increased time spent in screen-based activities across retirement was associated with small decrements in most mental health outcomes, which is consistent with previous cross-sectional research [50].

### Implications

The overall improvement in psychological health and wellbeing across retirement is a welcome finding. Given that the major time flows occurred from work into chores, sleep, quiet time and screen time, the net gain in psychological health and wellbeing appears to be driven by increases in sleep. The gains associated with increased sleep largely offset the decrement associated with increased screen time, whilst the increased time spent in chores was essentially neutral in terms of its impact on mental health. Physical activity was highlighted as the most important time-use behavior underpinning favorable changes in psychological wellbeing, yet increases in physical activity levels across retirement were modest. Findings highlight the need for programs which encourage increased participation in physical activity in retirement and avoiding increased screen time. This is particularly important given the well-recognized physical health consequences of physical activity and screen time, above and beyond the psychological health impacts examined in this study. Encouraging physical activity in this age group is a challenge, with low rates of participation in sports and exercise activities relative to younger adult age groups [51], and increased risk of injury [52]. Thus future efforts should focus on promoting physical activity which is both safe and appealing, noting that even modest increases may provide benefits.

### Strengths and limitations

This study used accepted, validated instruments to assess a wide range of mental health constructs. We also used a validated, reliable use-of-time recall measure which provided a high-resolution snapshot of how people used their time. Multiple days were sampled at each point, including weekends and weekdays, and reporting was subject to the constraint of the 24-h day. The use of compositional data analysis, adjusted for potentially confounding changes in income and health, allowed all time use domains to be considered simultaneously, avoiding errors associated with the assumption of independence when using traditional statistical approaches.

Study limitations included the use of self-report, which always carries a risk of recall and social desirability bias. However, the constraint of the 24-hour time-frame limits this risk. In addition, the sampling period represented only a fraction of the year, and may not have been representative. The study period was 12 months, which may not have allowed sufficient time for full adjustment to retirement. Many of our participants were still engaged with work activities post retirement, and we may have obtained different results for a non-working retirement sample. The sample size was relatively modest, and was not representative of Australian retirees, being somewhat wealthier and better educated [53]. They also atypical in that many they had well-paid positions of responsibility before retirement (54% professionals or associate professionals, and 16% managers). Post-retirement outcomes may have been different for people in physically demanding jobs. Most retired voluntarily for financial or lifestyle reasons, with only 12 being retrenched, and 5 citing illness. About half our participants were still engaged with work activities post-retirement, and we may have obtained different results with a non-working retirement sample. Previous studies [42, 54, 55] have identified multiple subgroups among retirees with different trajectories of wellbeing, self-efficacy and life satisfaction trajectories among retirees, depending on factors such as voluntariness of retirement and bridge work. Our sample may therefore be more typical of a subgroup than of the generality of retirees. While this study found no significant differences between men and women, studies with other populations and higher power may well identify differences both in how time use changes, and the relationship between time-use changes and health outcomes. Finally, because there was no non-retiring control group, we cannot be sure that these changes were entirely due to the sequelae of retirement and not to ageing per se.

### Conclusion

Life transitions provide both threats and opportunities for health-related behaviour change. Transitions can be pivotal moments when people consciously decide to adopt healthier lifestyles, or times when they fall into unhealthy behaviour patterns. If the longitudinal patterns found in this study reflect causal associations, it is possible that appropriate planning of use of time in retirement may improve mental health. Specifically, retirees should be encouraged to become more physically active, to sleep more, and to reduce screen time.

## Supporting information

S1 File Complete dataset. The complete dataset on which this analysis was based.
(XLSX)
